# Candidate gene polymorphisms and power athlete status: a meta-analytical approach

**DOI:** 10.1007/s13105-025-01071-0

**Published:** 2025-03-08

**Authors:** Gökhan İpekoğlu, Tuğba Çetin, Tülay Sırtbaş, Rabia Kılıç, Mustafa Odabaşı, Fahrettin Bayraktar

**Affiliations:** 1 Faculty of Sport Science, Ordu University, Ordu, Turkey; 2 Hasan Dogan Faculty of Sports Sciences, Karabuk University, Karabuk, Turkey

**Keywords:** AGTR2, FTO, GALNTL6, GNB3, MCT1, NOS3

## Abstract

Recent studies have focused on genetic polymorphisms that may influence athlete status. This meta-analysis aimed to investigate the association between athlete status and specific candidate genetic polymorphisms (*AGTR2 rs11091046, FTO rs9939609, GALNTL6 rs558129, GNB3 rs5443, MCT1 rs1049434, NOS3 rs2070744*). Only case–control studies collected from PubMed and Web of Science databases, published between 2009 and 2022, were included. A total of 23 studies were included in the meta-analysis according to the criteria of the research, and analyses were performed using random or fixed effects models. Effect size, odds ratio, or risk ratio were evaluated with a suitable 95% confidence interval. The results showed that the *GALNTL6 rs558129 T/T* genotype, *MCT1 rs1049434 T/T* genotype, and *NOS3 rs2070744 T* allele and *T/T* genotype were more prevalent in power athletes than in controls (p < 0.05). Conversely, the *GALNTL6 rs558129 C* allele, *C/C* genotype, and *AGTR2 rs11091046 C* allele and *C/C* genotype were more common in the control group. These findings indicate that some genetic polymorphisms may be important markers in athlete status and should be supported by future studies.

## Introductıon

Research on the impact of genetics on athletes has been increasing in recent years. Approximately 66% of athletes' athletic performance is associated with genetic factors, depending on the discipline [1]. Evidence in the literature suggests that genetic polymorphisms are linked to factors such as muscle fiber and fibril structure, influencing athletic performance [2]. When this effect is examined according to sports disciplines, variations in mitochondrial genes in endurance sports influence energy production and oxygen utilization, thereby determining an individual's endurance capacity and overall performance [3–5]. However, when it comes to power sports, genetic polymorphisms play a significant role in muscle growth, power production, and strength development [6]. In power athletes, specific genetic variations can influence the distribution of muscle fiber types, muscle protein synthesis, and muscle contraction speed, thereby shaping individual performance [7]. This influence on muscle protein synthesis, in particular, can affect an athlete's capacity for muscle growth and strength gain. Similarly, by impacting the production and function of proteins that determine contraction speed and power.

Studies on genetic variants related to power athlete status provide a fundamental framework for understanding the performance and physiological characteristics of athletes [8, 9]. In this field, the *ACTN3* and *ACE* polymorphisms are among the most extensively investigated variants, with their effects on power performance well-supported by findings [10–14]. Additionally, other polymorphisms such as *PPARGC1A, AGT, CKMM, HIF1A*, *AMPD1, IL-6, CPNE5* and *PPARA* have been identified as genetic variants that play essential roles in the physiological profiles of power athletes, contributing to critical biological processes. Various meta-analyses have suggested that these variants may impact key physiological mechanisms, including muscle function, oxygen transport capacity, inflammatory responses, and energy metabolism [2, 15–21]. However, recent advancements in the field of genetics have accelerated research in sports genetics, prompting researchers to more closely examine how athletes' genetic makeup may influence athletic performance [22–24]. Recent research suggests that, beyond *ACE*, *ACTN3*, and other frequently studied genes, a broader spectrum of genetic variants may contribute to advantageous physiological traits in power athletes [1]. Some of the variants that have recently been studied in relation to the power-athlete relationship include Angiotensin II receptor, type 2 *rs11091046* (*AGTR2*), Fat Mass and Obesity-Associated *rs9939609* (*FTO*), N-acetylgalactosaminyltransferase-like 6 enzyme *rs558129* (*GALNTL6*), Guanine nucleotide-binding protein beta polypeptide 3 *rs5443* (*GNB3*), Monocarboxylate transporter 1 *rs1049434* (*MCT1*), Nitric oxide synthase *rs2070744* (*NOS3*) genes. Variants like *AGTR2*, *FTO*, *GALNTL6*, *GNB3*, *MCT1*, and *NOS3* contribute to essential processes such as oxygen transport, metabolic efficiency, and cellular signaling, all of which are crucial for power sports but have been underexplored. Examining these less-studied polymorphisms provides a novel perspective on the genetic foundations of power athlete status, advancing insights into the physiological traits that support elite power capabilities.

The A > C polymorphism (*rs11091046*) of the *AGTR2* gene, located at Xq22-q23, is a component of the renin-angiotensin system (RAS), which is unquestionably the most important hormonal system in the control of the circulatory system and blood pressure [25]. Within this system, angiotensin II acts through two receptors: *AGTR1*, primarily responsible for vasoconstriction, and *AGTR2*, which influences cellular growth and differentiation [26, 27]. This dual action can enable the RAS to influence muscle growth and vascular responses, potentially linking it to performance factors in power-focused athletic activities. This effect on muscle growth can also help shape the muscle fiber types that contribute to athletic performance, such as strength and explosiveness. In power athletes, muscle fiber type II (fast-twitch fibers) plays a critical role in generating strength and explosiveness, which are essential for athletic performance in power-based activities [28]. To increase the number of known genetic determinants for muscle fiber types, a study associating 15 relevant polymorphisms was conducted [29]. Among the 15 candidate polymorphisms, only *rs11091046* was shown to be associated with muscle fiber type composition. This polymorphism was reported to explain 15.2% of the variation in muscle fiber type composition in the Vastus Lateralis muscle. Moreover, some studies suggest that the *A* allele of *rs11091046* may be associated with traits that could be beneficial for power athletes, potentially influencing factors such as muscle composition and performance in power-based activities [9, 29].

The *FTO* gene, located at position 16q12.2 on chromosome 16, is expressed in the hypothalamic region of the brain, which is central to the control of food intake. Considering the intronic position of *A/T* (*rs9939609*) polymorphism, *FTO* plays a transcriptional regulatory role in upregulating or downregulating its expression [30]. Research has examined the association of the *FTO* gene with obesity, focusing on its role in metabolic regulation and energy balance [31, 32]. Building on this, obesity-associated genetic variations in the *FTO* gene have been shown to influence the differentiation of fat cells. These variations may promote the expression of genes associated with the development of white adipocytes, which store energy, rather than beige adipocytes, which dissipate energy as heat. This shift in fat cell phenotype supports energy conservation and increases fat storage, providing a possible mechanism by which *FTO* variations contribute to obesity-related traits [33]. Previous genome-wide association studies highlighted the link between *FTO T/A* polymorphism and body mass, with the *A* allele being associated with increased weight, waist circumference, and subcutaneous fat [31, 32]. In power athletes, this polymorphism may influence body composition and energy balance, potentially enhancing performance by promoting lean muscle mass while also affecting fat storage. Some studies have suggested that the *A* allele of the *FTO* gene is more prevalent in strength athletes, particularly those involved in power and combat sports, and is associated with higher body weight [34, 35]. However, other research has not found a significant relationship between this polymorphism and power athlete status [2, 30, 36]. Therefore, understanding the role of this polymorphism in determining athlete status is crucial.

The enzyme N-acetylgalactosaminyltransferase-like 6 (*GALNTL6*), encoded by the *GALNTL6* gene, plays a significant role in regulating short-chain fatty acids in the intestinal microbiome and their resynthesis [37]. The *GALNTL6* (*rs558129 C* > *T*) gene, located at 4q34.1, consists of 21 exons and exhibits predominant expression in adult tissues such as the skeletal muscles, brain, cerebellum, spinal cord, and testes [38]. Initially, Rankinen et al. [39] analyzed endurance athletes from seven countries under the GAMES consortium. While no universal genetic variant was identified, meta-analysis results highlighted the statistical significance of the *rs558129 C/T* polymorphism in the *GALNTL6* gene. Notably, endurance athletes were found to be 23% more likely to carry the *C* allele compared to controls. Building on these findings, Ramírez et al. [40] investigated the role of the *GALNTL6 C/T* polymorphism in power athlete status. Their study revealed that the *T* allele was associated with improved anaerobic performance. Additionally, they observed a higher frequency of the *C* allele among long-distance swimmers, suggesting a potential influence of this polymorphism on performance traits specific to different athletic disciplines. However, despite these indications that the polymorphism may influence power athlete status, further studies are necessary to better understand its full impact. Consequently, conducting a meta-analysis in this context is essential, as it can provide more robust evidence and clarify the role of the *GALNTL6* gene in power sport performance across different studies.

The *GNB3* gene is situated on the short arm (p) 21.3 region of chromosome 12. This gene is responsible for encoding the beta-3 subunit of heterotrimeric G proteins, which play a crucial role in facilitating signal transmission between G protein-coupled receptors and intracellular effectors in nearly all human body cells [41]. The gene exhibits a polymorphism at position 825 (*rs5443*), involving a substitution of cytosine (*C*) with thymine (*T*). The *825 T* allele, corresponding to the shorter isoform, is linked to increased G-protein activity [42]. Although the *GNB3 C825T* polymorphism is often linked to obesity and increased hypertension, it is also considered a potential candidate gene for physical performance [43, 44]. For instance, Faruque et al. [45]discovered an inverse association between the *T* allele and VO2 peak, employing a recessive model of inheritance. Their study indirectly connected the *GNB3 C825T* variant to VO2 peak by its influence on heart rate variability. In a similar context, another study investigated the distribution frequencies of the *T/T* genotype of the *GNB3 C825T* polymorphism across endurance athletes, power athletes, and control groups. The findings revealed that the *T/T* genotype frequency was higher among endurance athletes [46]. However, studies exploring the association between this polymorphism and power athlete status remain limited. Notably, a meta-analysis conducted by Weyerstraß et al. [2], which examined the relationship between genetic polymorphisms and power athlete status, found no significant association between the *GNB3 C825T* polymorphism and power athletes. Despite these mixed findings, our decision to include this gene in our meta-analysis stems from its potential to contribute to a broader understanding of genetic influences on athletic performance. By re-evaluating its role within a comprehensive analytical framework, our study aims to clarify the relevance of this polymorphism, particularly in the context of power-oriented sports, where genetic determinants are still being elucidated.

The *MCT1* gene, alternatively known as *SLC16A1* and located at position 1p12, encodes the *MCT1* protein, which is part of metabolic genes expressed in skeletal muscle and regulated by *PGC-1α* [47, 48]. The muscle contractions occurring, especially during intense exercises, lead to the production of lactate [49]. It has been demonstrated that the expression of skeletal muscle *MCT1* is correlated with the rate constant of net blood lactate removal after a 1-min all-out test and with fatigue indexes [50]. The *MCT* isoforms in skeletal muscles, namely *MCT4* and *MCT1*, play a crucial role in facilitating lactic acid release from glycolytic muscles for ATP production. Additionally, *MCT1* is essential for the uptake of lactate produced by white muscle fibers into myocytes, supporting its oxidation in the heart and red skeletal muscle, where lactate serves as a primary respiratory fuel [51]. Recent studies suggest an association between the *T1470A* polymorphism (*rs1049434 A/T*) of *MCT1* and athletic performance, power athlete status, and physiological phenotypes [52, 53].

Numerous studies have emphasized the significant role of nitric oxide (NO) in facilitating glucose uptake in human skeletal muscles during exercise and explained its intricate involvement in modulating oxygen consumption within the skeletal muscles [54, 55]. In their study on eNOS (–/–) mice, Le Gouill et al., [56] observed that mice lacking endothelial nitric oxide synthase (eNOS) exhibited lower oxygen consumption and impaired mitochondria compared to normal mice. In this case, it is believed that the negative impact on the beta-oxidation process could lead to a decrease in energy production. Since this condition is regulated by the *NOS3* gene responsible for the production of NO, it is believed to have potential effects on athletes' athletic performance. The *NOS3* gene located on chromosome 7 (7q36) contains two isoforms, predominantly nNOS found in skeletal muscles and generally eNOS located in endothelial cells [57]. The polymorphisms in the *NOS3 gene, −786 T/C* (*rs2070744*) in the promoter region and *G894T* (Glu298Asp) single nucleotide polymorphisms (SNPs) in exon 7, are the most extensively studied and functionally associated variants [58]. In various studies, the *NOS3 −786 T/C* variant has been proposed as a candidate gene that could play a role in the relationship between athlete status and physical performance [59]. In a recent study examining genetic markers that could be candidates for power athlete status, the *T* allele of the *NOS3 786 T/C* (*rs2070744*) variant emerged as one of the most promising genetic markers [1].

The importance of conducting a meta-analysis in this field stems from the need to clarify and integrate findings on the genetic polymorphisms associated with muscle strength and power. While a substantial number of studies have identified potential genetic markers related to these attributes, the consistency of such associations remains unclear. By synthesizing data across multiple studies, a meta-analysis can offer robust evidence on whether each specific single nucleotide polymorphism (SNP) truly correlates with strength and power traits, beyond the findings of individual studies. This approach not only enhances the reliability of observed associations but also facilitates a comprehensive understanding of the functional roles of these genes in athletic performance. Therefore, this meta-analysis aims to clarify the relationship between *AGTR2 rs11091046, FTO rs9939609, GALNTL6 rs558129*, *GNB3 rs5443, MCT1 rs1049434, and NOS3 rs2070744* polymorphisms and power athlete status. By addressing the limitations of previous studies, this work will provide a valuable reference for researchers and contribute significant insights into the genetic predisposition for power athlete status.

## Methods

### Search strategy

A comprehensive search was conducted in electronic databases such as PubMed and Web of Science to identify studies on *AGTR2, FTO, GALNTL6, GNB3, MCT1*, and *NOS3* gene polymorphisms and their association with power athlete status. The population examined in the research included power athletes for case groups and sedentary individuals for control participants, with studies covering the period from the first research in 2009 to the latest in 2022. The search strategy used the following keywords: "*AGTR2* polymorphism," "*FTO* polymorphism," "*GALNTL6* polymorphism," "*GNB3* polymorphism," "*MCT1* polymorphism," "*NOS3* polymorphism," along with "athlete," and "power." The search was limited to studies published in English and containing original research articles. The "related articles" feature in electronic databases was used to identify additional studies that may have been missed initially. The reference lists of relevant studies were also examined to identify potentially missed studies. Additionally, contact was made with experts in the field to inquire about any additional studies that may not have been identified in the search. During the database search using the keywords, priority was given to studies with titles potentially relevant to the topic of meta-analysis, and these studies were recorded. Subsequently, the abstracts of the studies were reviewed, and studies that did not meet the inclusion criteria were excluded from the content.

### Inclusion criteria

The inclusion criteria for the study were as follows: (1) Investigating the relationship between *AGTR2* (*rs11091046*), *FTO* (*rs9939609*), *GALNTL6* (*rs558129*), *GNB3* (*rs5443*), *MCT1* (*rs1049434*), *NOS3* (*rs2070744*) gene polymorphisms and power athlete status, (2) Including both a case and a control group in the study, (3) Providing sufficient data to calculate the odds ratio and 95% confidence interval, (4) Articles having a Newcastle–Ottawa Scale score of more than 5, and (5) Articles being in English full text. Exclusion criteria included: (1) Studies classified as review articles, (2) Studies classified as case reports, (3) Studies that did not provide sufficient data to calculate the risk ratio and did not include 95% confidence intervals, (4) Studies with a Newcastle–Ottawa Scale score of less than 6, and (5) Studies not having English full text.

### Data extraction

Two review experts independently extracted data from the identified studies using a standardized data extraction form. Data points were collected from each study: (1) Study characteristics (study design, sample size, population characteristics), (2) Details regarding gene polymorphisms (genotyping, allele frequencies), and (3) Results of the relationship between *AGTR2, FTO, GALNTL6, GNB3, MCT1*, and *NOS3* gene polymorphisms and power athlete status (e.g., odds ratios).

### Quality assessment

**Fig. 1:**
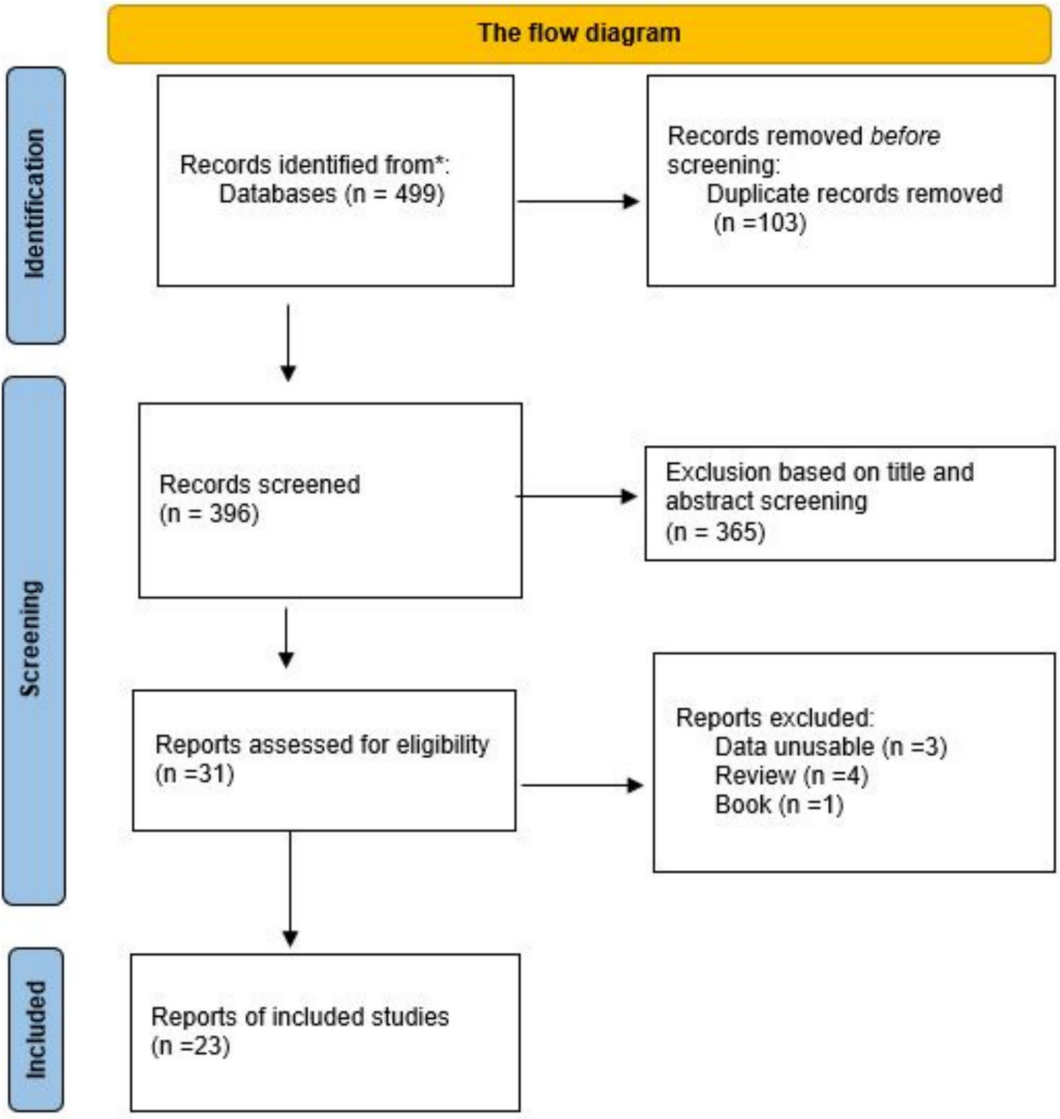
The flow diagram of included/excluded studies

**Fig. 2:**
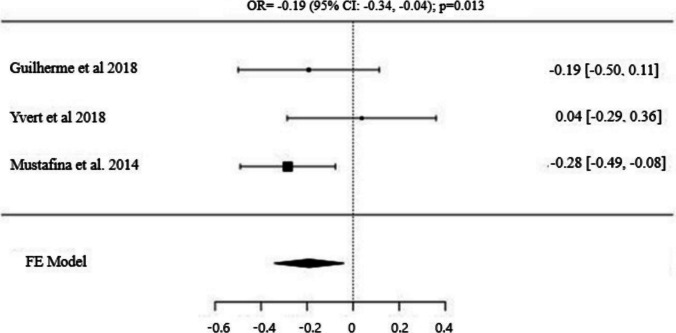
Forest Plot for AGTR2 (rs11091046) C/A Alleles: Odds Ratio (95% CI) with C as the Major Allele

**Fig. 3:**
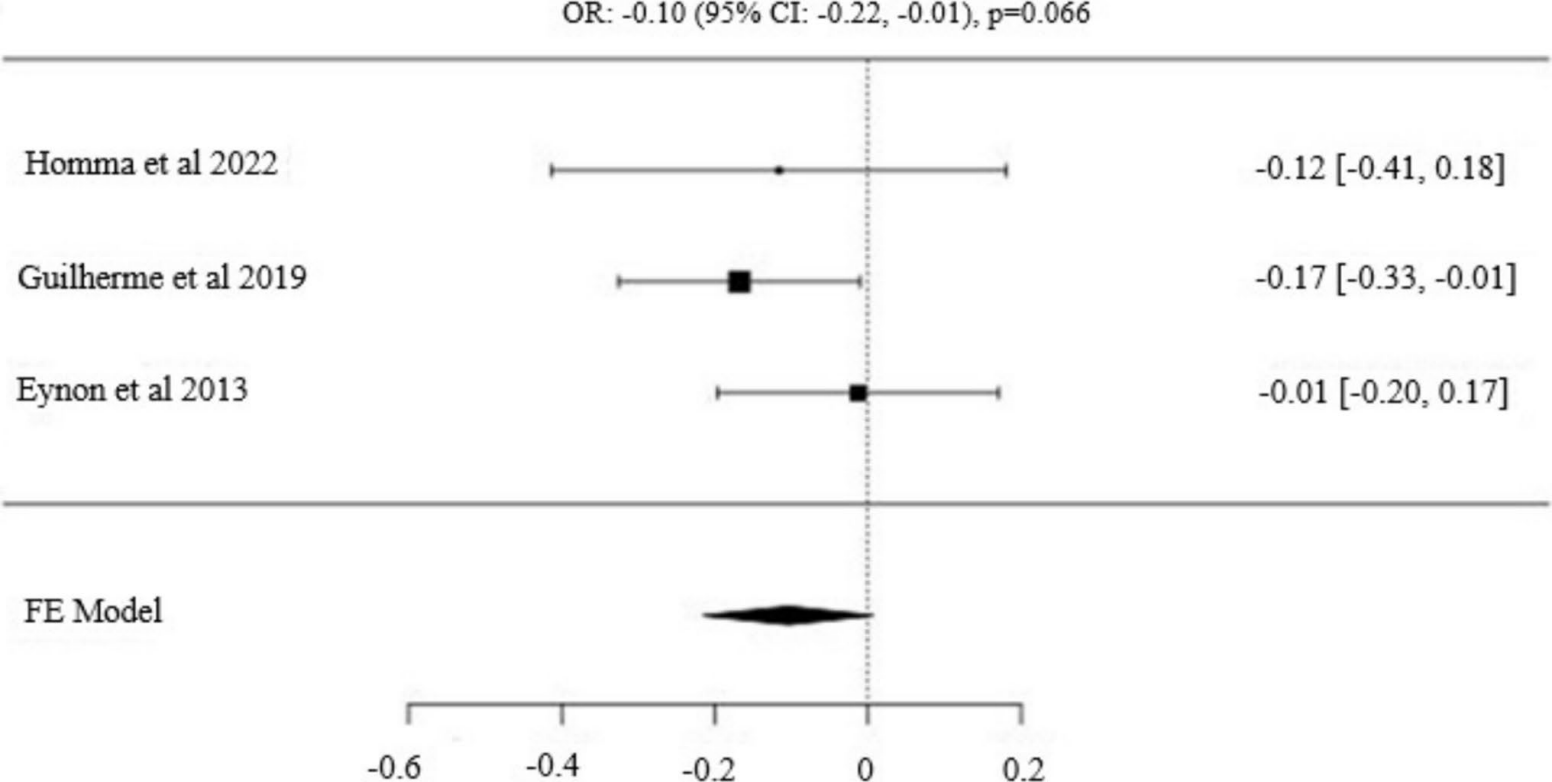
Forest Plot for *FTO* (*rs9939609*) *T*/*A* Alleles: Odds Ratio (95% CI) with T as the Major Allele

**Fig. 4:**
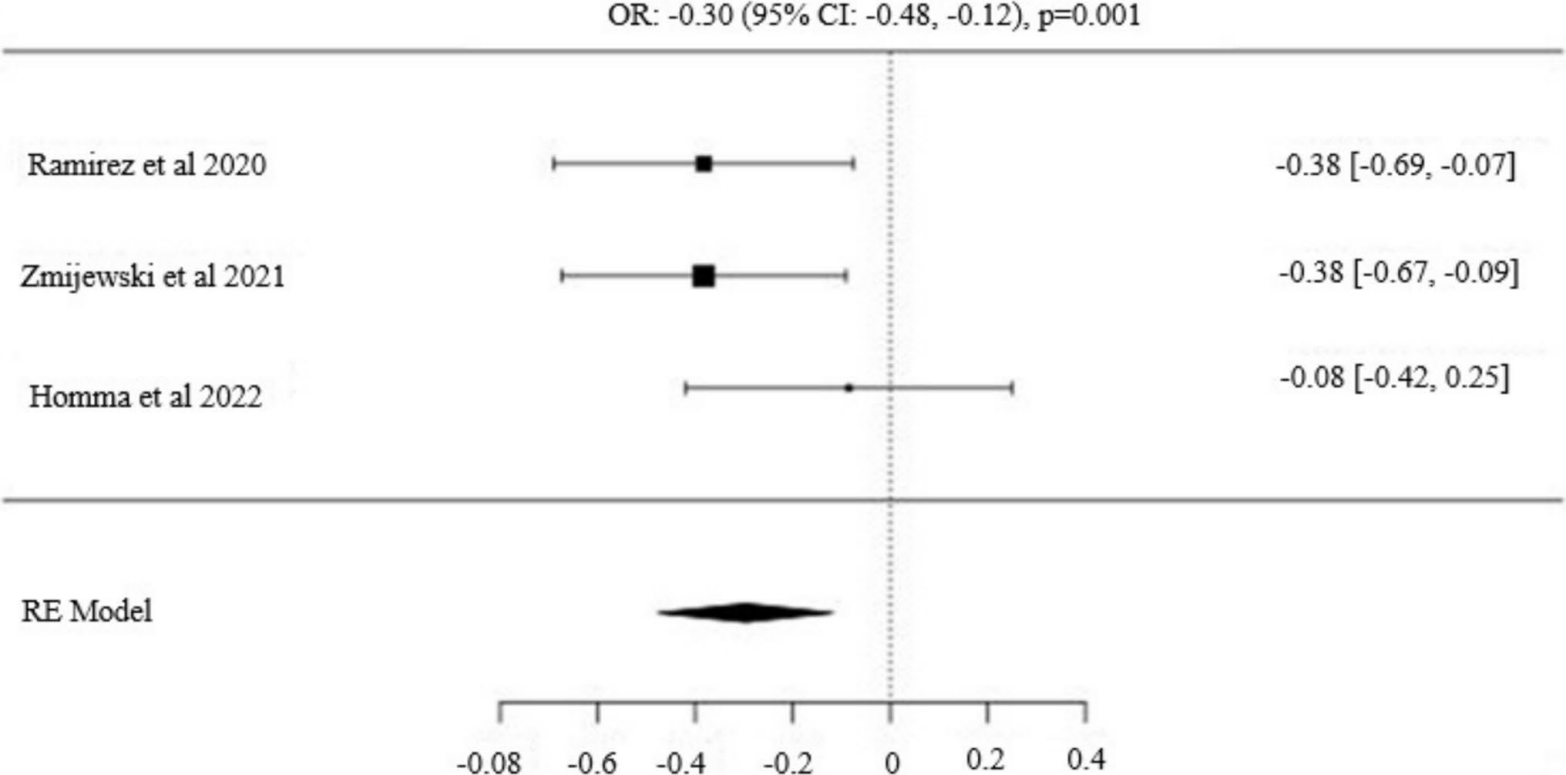
Forest Plot for *GALNTL6* (*rs558129*) *C/T* Alleles: Odds Ratio (95% CI) with C as the Major Allele

**Fig. 5:**
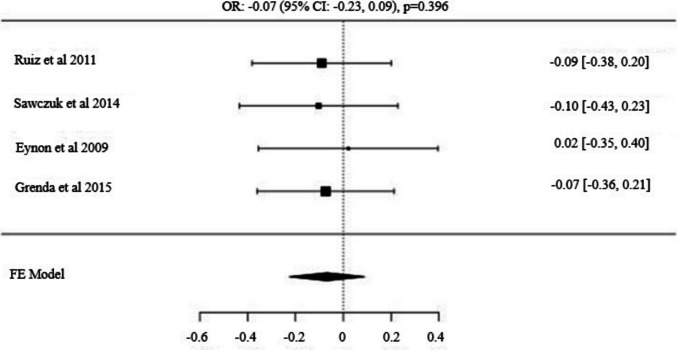
Forest Plot for *GNB3* (*rs5443*) *C/T* Alleles: Odds Ratio (95% CI) with C as the Major Allele

**Fig. 6:**
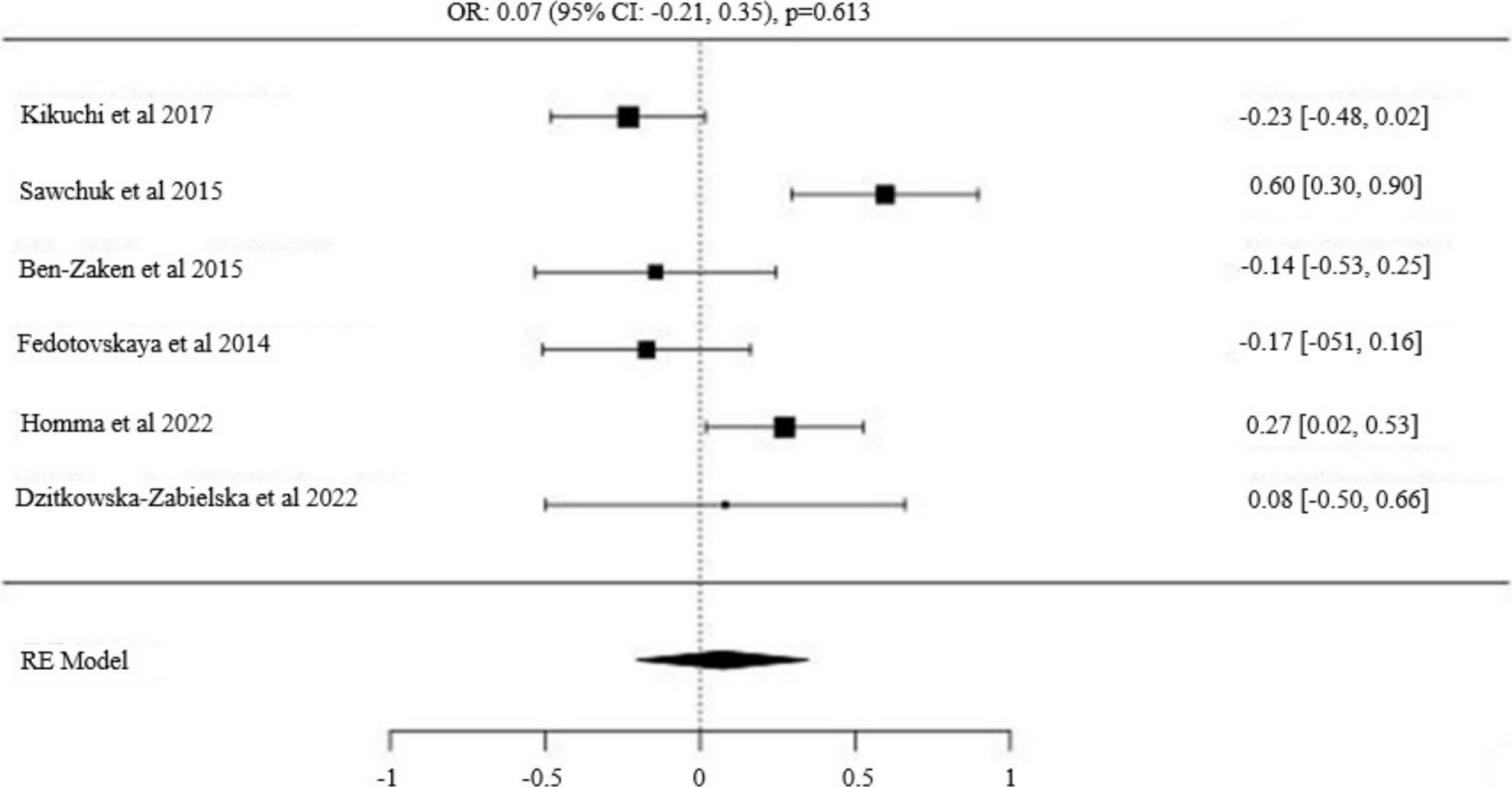
Forest Plot for *MCT1* (*1,049,434*) *A/T* Alleles: Odds Ratio (95% CI) with T as the Major Allele

**Fig. 7:**
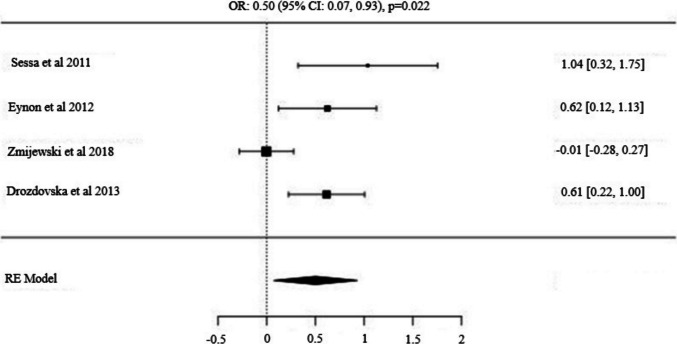
Forest Plot for *NOS3* (*rs2070744*) *T/C* Alleles: Odds Ratio (95% CI) with T as the Major Allele

**Fig. 8:**
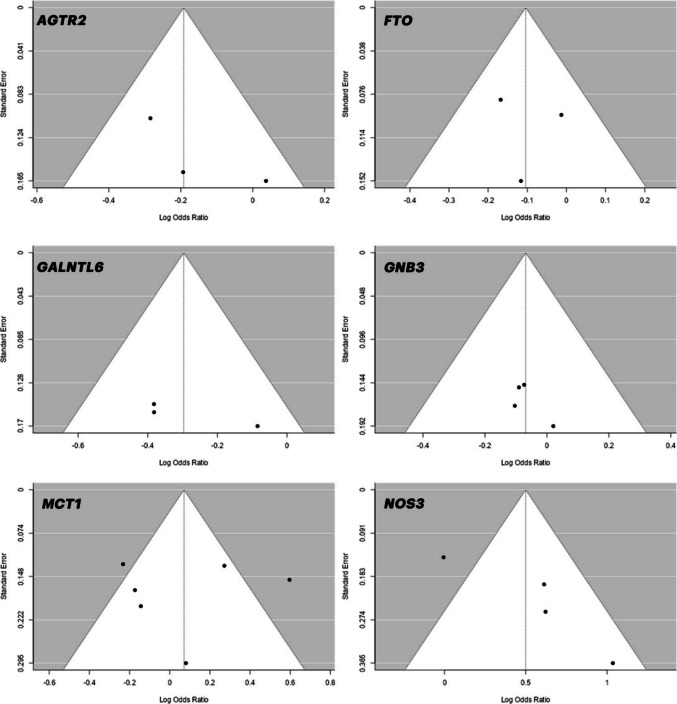
Major Allele-Based Funnel Plots for *AGTR2, FTO, GALNTL6, GNB3, MCT1, NOS3* Polymorphisms

The Newcastle–Ottawa Scale (NOS) was used to assess the quality of the studies. The scale was applied by two different authors, and any differences in interpretation between the authors were resolved through discussion. Each study was evaluated based on eight items on the NOS, divided into three groups: selection of study groups (four items), comparability of groups (one item), and determination of outcome of interest for cohort studies (three items). Each item was given a maximum of one point, and for cohort studies, a maximum of one star was given for each item related to determining the outcome of interest in study groups. Studies with 6 points or higher were considered to meet the criteria for sufficient quality and were included in the study (Figs. 1, 2, 3, 4, 5, 6, 7 and 8).

### Statistical analysis

Statistical analysis involved the use of random effects or fixed effects models depending on the results of heterogeneity tests for the data. The presence of heterogeneity was assessed using the Cochran Q test and the I2(%) statistic [60]. Publication bias was assessed using funnel plots and the Egger test, while effect size was calculated as odds ratios or risk ratios with 95% confidence intervals. Due to the inability to identify three or more studies for sub-dimension analysis (e.g., gender, race), sub-dimensions were not included in the meta-analysis. In this study, a case–control design was used to examine the distribution of polymorphisms between power athletes and sedentary control individuals. Statistical analysis was performed using Jamovi 2.3.

## Result

This meta-analysis involved a search conducted using the keywords identified in the PubMed and Web of Science databases, resulting in the identification of 499 articles. After reviewing titles, abstracts, and full texts, 23 articles were deemed suitable for inclusion in this meta-analysis. Factors such as the absence of a control group, the lack of a group consisting solely of power athletes, and unclear allele/genotype data were considered restrictive factors, and articles containing these factors were not included in the meta-analysis.

This table provides descriptive data for all the articles included in the meta-analysis, including the authors of the study, the year of the study, the ethnicities of individuals in the control and power groups, the genes studied, and the number of participants in the study. Additionally, Table 1 presents the results of the Newcastle–Ottawa Scale used to evaluate the quality of the study.

**Table 1:** Descriptive Data of Included Studies

Study	Race	Gene Names (rs number)	Total Athletes	Total Controls	Newcastle–Ottawa Scale			
					Sel	Com	Exp	Total
Kikuchi et al. 2017	A	*MCT1* (*rs1049434*)	199 (Wrestlers)	649	4	2	3	9
Sawczuk et al. 2015	C	*MCT1* (*rs1049434*)	100 (Runners, jumpers, powerlifters, throwers, weightlifters)	621	4	1	3	8
Ben-Zaken et al. 2015	C	*MCT1* (*rs1049434*)	107 (Sprinters, jumpers, swimmers)	128	4	1	3	8
Fedotovskaya et al. 2014	C	*MCT1* (*rs1049434*)	91 (Heptathletes, runners, kettlebell lifters, weightlifters)	467	4	1	3	8
Homma et al. 2022	A	MCT1 (*rs1049434*) *FTO* (*rs9939609*) *GALNTL6* (*rs558129*)	192 (Weightlifters)	416	4	2	3	9
Guilherme et al. 2018	H	*AGTR2* (*rs11091046*)	124 (Swimmers, gymnasts, jumpers / throwers, runners, cyclists, canoe sprinters, weightlifters)	241	4	2	3	9
Yvert et al. 2018	A, C	*AGTR2* (rs11091046)	82 (Sprinters, Jumpers, throwers)	1182	4	1	3	8
Mustafina et al. 2014	C	*AGTR2* (*rs11091046*)	261 (Sprinters)	589	4	2	3	9
Eynon et al. 2013	C	*FTO* (*rs9939609*)	285 (Sprinters, weightlifters, jumpers, skaters, swimmers)	1416	4	2	3	9
Drozdovska et al. 2013	C	*NOS3* (*rs2070744*)	90 (Runners, swimmers, jumpers, throwers)	321	4	1	3	8
Eynon et al. 2012	C	*NOS3* (*rs2070744*)	53 (Sprinters, jumpers, throwers)	100	4	1	3	8
Zmijewski et al. 2018	C	*NOS3* (*rs2070744*)	147 (Swimmers)	379	4	2	3	9
Ruiz et al. 2011	C	*GNB3* (*rs5443*)	134 (Sprinters)	340	4	1	3	8
Sawczuk et al. 2014	C	*GNB3* (*rs5443*)	100 (Runners, powerlifters, weightlifters, throwers, jumpers)	354	4	1	3	8
Eynon et al. 2009	H	*GNB3* (*rs5443*)	81 (Sprinters)	234	4	2	3	9
Díaz Ramírez et al. 2020	C	*GALNTL6* (*rs558129*)	173 (Weightlifters, sprinters, runners)	201	4	2	3	9
Zmijewski et al. 2021	C	*GALNTL6* (*rs558129*)	147 (Swimmers)	379	4	2	3	9
Guilherme et al. 2019	C, H	*FTO* (*rs9939609*)	420 (Artistic gymnasts, jumpers/throwers, runners, cyclists, canoe speed, jiu-jitsu, judo, karate, kung Fu, martial arts, muay thai, taekwondo, wrestlers)	1406	4	2	3	9
Grenda et al. 2015	C	*GNB3* (*rs5443*)	147 (Swimmers)	379	4	1	3	8
Dzitkowska-Zabielska et al. 2022	C	*MCT1* (*rs1049434*)	53 (Sprinter, weightlifters, powerlifters)	41	4	1	3	8
Sessa et al. 2011	C	*NOS3* (*rs2070744*)	29 (Sprinters, swimmers, volleyball)	38	4	1	3	8

A, Asian; C, Caucasion; E, European; H, Hispanic; Sel, Selection; Com, Comparability; Exp, Explosure.

Initially, a total of 499 studies were identified, but only 23 met the necessary criteria and were included in the meta-analysis. Table 2 presents the overall analyses of the association between the allele-based odds ratio (OR) of the investigated genes and power athletes, along with information on heterogeneity and publication bias. The table includes data on the genes *AGTR2* (*rs11091046*), *FTO* (*rs9939609*), *GALNTL6* (*rs558129*), *GNB3* (*rs5443*), *MCT1* (*rs1049434*), and *NOS3* (*rs2070744*). It provides allele-based ORs, confidence intervals, heterogeneity statistics (I2 and Q), and publication bias assessment (Egger's test).

**Table 2:** Overall analyses of the association between the allele based OR of the investigated genes and power athletes

	Meta Analysis							Heterogeneity			Publ. Bias
	Gene	n	MA	OR	Lower CI	Upper CI	P	Heterogeneity I^2^	Heterogeneity Q (df)	p-Value of Q	Egger’s
**Allele based OR** **(95%Cl)**	***AGTR2 (rs11091046)***	3	C	−0.19	−0.34	−0.04	**0.013**	25.86%	2.698	0.260	0.016
	***FTO (rs9939609)***	3	T	−0.10	−0.22	−0.01	0.066	0%	1.578	0.454	0.042
	***GALNTL6 (rs558129)***	3	C	−0.30	−0.48	−0.12	**0.001**	0.76%	2.174	0.337	0.001
	***GNB3 (rs5443)***	4	C	−0.07	−0.23	0.09	0.396	0%	0.283	0.963	0.210
	***MCT1 (rs1049434)***	6	T	0.07	−0.21	0.35	0.613	76.77%	23.082	0.001	0.135
	***NOS3 (rs2070744)***	4	T	0.50	0.07	0.93	**0.022**	73.73%	12.622	0.006	0.001
**DM based OR** **(95%Cl)**	***AGTR2 (rs11091046)***	3	C	−0.38	−0.62	−0.14	**0.002**	33.47%	3.006	0.222	0.002
	***FTO (rs9939609)***	3	T	−0.13	−0.29	0.02	0.094	0.91%	2.018	0.365	0.058
	***GALNTL6 (rs558129)***	3	C	−0.32	−0.55	−0.09	**0.005**	11.22%	2.253	0.324	0.002
	***GNB3 (rs5443)***	4	C	−0.12	−0.34	0.10	0.275	0%	0.407	0.939	0.136
	***MCT1 (rs1049434)***	6	T	0.34	−0.10	0.59	**0.006**	50.67%	10.136	0.071	0.009
	***NOS3 (rs2070744)***	4	T	0.73	0.12	1.34	**0.018**	73.31%	11.391	0.010	0.001
**RM based OR** **(95%Cl)**	***AGTR2 (rs11091046)***	3	C	0.02	−0.65	0.69	0.959	79.93%	7.475	0.024	0.332
	***FTO (rs9939609)***	3	T	0.13	−0.08	0.35	0.232	0%	0.252	0.882	0.134
	***GALNTL6 (rs558129)***	3	C	0.54	0.11	0.96	**0.014**	0%	0.683	0.711	0.011
	***GNB3 (rs5443)***	4	C	0.03	−0.32	0.40	0.844	0%	2.064	0.559	0.496
	***MCT1 (rs1049434)***	6	T	0.01	−0.37	0.40	0.949	76.6%	21.780	0.001	0.430
	***NOS3 (rs2070744)***	4	T	−0.20	−0.58	0.17	0.284	41.06%	5.090	0.165	0.048

*MA*, major allele, *OR*, odds ratio, *CI*, confidence interval, *DM*, dominant model, *RM*, recessive model.

## The *AGTR2* (*rs11091046*) allele and genotype distribution (Power vs Control)

Three studies analyzed the distribution of the *AGTR2* (*rs11091046*) SNP in power athletes, including 467 power athletes and 2012 control subjects [29, 61, 62]. The frequency of the major allele (*C*) was higher in the control group compared to power athletes, and this difference was statistically significant [OR: −0.19 (95% CI: −0.34, −0.04); p = 0.013]. Similarly, the dominant model (*C/C vs. C/A* + *A/A*) was more prevalent in the control group than in power athletes, with a statistically significant difference [OR: −0.38 (95% CI: −0.62, −0.14); p = 0.002]. In contrast, no significant difference was observed in the recessive model (*AA vs. CA* + *CC*) between the groups [OR: 0.02 (95% CI:−0.65, 0.69); p = 0.959], indicating no clear association between the recessive genotype and power athlete status.

### The *FTO* (*rs9939609*) allele and genotype distribution (Power vs Control)

A total of 897 power athletes and 3238 control subjects were analyzed across three studies examining the *FTO* gene polymorphism [34, 36, 53]. No significant difference was observed in the frequency of the major allele (*T*) between power athletes and controls [OR: −0.10 (95% CI: −0.22, −0.01), p = 0.066]. Similarly, the dominant model (*T/T vs. T/A* + *A/A*) showed no statistically significant difference in frequency between the two groups [OR: −0.13 (95% CI: −0.29, −0.02), p = 0.094]. Additionally, no significant difference was found in the recessive model (*A/A* vs. *T/A* + *T/T*) [OR: 0.13 (95% CI: −0.08, 0.35), p = 0.232].

### The *GALNTL6* (*rs558129*) allele and genotype distribution (Power vs Control)

Three studies examining the *GALNTL6* gene polymorphism included 512 power athletes and 996 control subjects [37, 40, 53]. The frequency of the major allele (*C*) was significantly higher in the control group compared to power athletes [OR: −0.30 (95% CI: −0.48, −0.12), p = 0.001]. Similarly, the dominant model (*C/C vs. C/T* + *T/T*) was more prevalent in the control group, with a significant difference observed [OR: −0.32 (95% CI: −0.55, −0.09), p = 0.005]. Conversely, the recessive model (*T/T vs. C/T* + *C/C*) showed a significantly higher frequency in power athletes compared to controls [OR: 0.54 (95% CI: 0.11, 0.96), p = 0.014].

### The *GNB3* (*rs5443*) allele and genotype distribution (Power vs Control)

Four studies examining the *GNB3* gene polymorphism in power athletes included a total of 462 power athletes and 1,307 control subjects [44, 46, 63, 64]. No significant difference was observed in the major allele (*C*) distribution frequency between power athletes and controls [OR: −0.07 (95% CI: −0.23, 0.09), p = 0.396]. Similarly, neither the dominant model (*C/C vs. C/T* + *T/T*) [OR: −0.12 (95% CI: −0.34, 0.10), p = 0.275] nor the recessive model (*T*/*T vs. C*/*T* + *C*/*C*) [OR: 0.03 (95% CI: −0.32, 0.40), p = 0.844] showed significant differences in distribution frequencies.

### The *MCT1* (*rs1049434*) allele and genotype distribution (Power vs Control)

Six studies meeting the inclusion criteria for the *MCT1* gene polymorphism included a total of 742 power athletes and 2,322 control subjects [48, 49, 52, 53, 65, 66]. The analysis of the *MCT1* (*rs1049434*) SNP revealed no significant difference in the frequency of the major *T* allele between power athletes and controls [OR: 0.07 (95% CI: −0.21, 0.35), p = 0.613]. The recessive model (*T/T vs. T/A* + *A/A*) also showed no significant difference in distribution frequencies between the groups [OR: 0.01 (95% CI: −0.37, 0.40), p = 0.949]. However, a significant difference was observed in the dominant model (*T/T vs. T/A* + *A/A*), with this genotype being more frequent in power athletes compared to the controls [OR: 0.34 (95% CI: −0.10, 0.59), p = 0.006].

### The *NOS3 (rs2070744)* allele and genotype distribution (Power vs Control)

Four studies examining the distribution of *NOS3* (*rs2070744*) SNP in power athletes included a total of 319 power athletes and 838 control subjects [57, 67–69]. The analysis demonstrated a significant difference in the frequency of the major *T* allele, with a higher prevalence observed in power athletes compared to controls [OR: 0.50 (95% CI: 0.07, 0.93), p = 0.022]. Similarly, the dominant model (*T/T vs. T/C* + *C/C*) showed a significantly higher frequency of the *T/T* genotype in power athletes compared to controls [OR: 0.73 (95% CI: 0.12, 1.34), p = 0.018]. In contrast, the recessive model (*C/C vs. T/C* + *T/T*) did not reveal significant differences [OR: −0.20 (95% CI: −0.58, 0.17), p = 0.284], suggesting that the dominance of the *T* allele and the *T/T* genotype may contribute to power athlete status.

## Dıscussıon

The main objective of this study is to investigate the allele and genotype distribution frequencies of six candidate genes believed to be associated with the status of being a power athlete through a meta-analysis approach. This meta-analysis is based on a detailed examination of 23 scientific studies published between 2009 and 2022. The study focuses on the analysis and interpretation of the genotypic distributions of *AGTR2 rs11091046, FTO rs9939609, GALNTL6 rs558129, GNB3 rs5443, MCT1 rs1049434, and NOS3 rs2070744* polymorphisms in relation to power athlete statuses.

It is well-known that muscle strength is a crucial attribute for athletes, particularly those involved in power sports, to achieve optimal and peak performance [21]. In the significant role of muscle strength, the contribution of Angiotensin II, the main effector molecule of the Renin-Angiotensin System (RAS), holds great importance. Acting through receptors *AGTR1* and *AGTR2*, Angiotensin II helps regulate blood vessel tone and supports muscle growth, serving as a powerful vasoconstrictor and muscle growth factor [9, 26]. These interactions may contribute to athletes enhancing muscle strength for achieving optimal performance. However, muscle fiber type, size, and the proportion of different fiber types that athletes possess can be partially determined by genotypes. Thus, genotypes associated with power sports can be identified and may prove beneficial in optimizing training programs [2]. In a study investigating the relationship between muscle fiber composition and *AGTR2 A/C* (*rs11091046*) polymorphism among candidate genes, the *C* allele was associated with a higher proportion of slow-contracting muscle fibers, while the *A* allele was linked to a higher proportion of fast-contracting fibers [29]. According to the findings of the study, the frequency of the *A* allele was found to be higher in female power athletes, particularly in comparison to the control group. Similarly, in line with the results of the study conducted by Guilherme et al. [61], it was revealed that the minor allele (*A*) distribution frequencies of the *AGTR2* (*rs11091046*) gene polymorphism in power athletes, especially at the international level, were higher than those in the control group and endurance athletes. In contrast to these findings, Yvert et al. [62] observed a higher frequency of the *C* allele in male sprint/power athletes in their study. In contrast, the results of this meta-analysis, which provides a comprehensive examination of the *AGTR2 A/C* (*rs11091046*) polymorphism, revealed significant differences in the distribution frequencies of both the major allele (*C*) and the dominant model (*C/C*). These findings indicate that the *C* allele and *C/C* genotype are significantly more prevalent in the control group compared to power athletes. This suggests that the likelihood of carrying the *C* allele or the *C/C* genotype may be lower among power athletes, potentially indicating a less prominent role for this genetic variant in traits associated with power athletic performance. Moreover, unlike some previous studies, no significant relationship was observed between the *A* allele and power athlete status, further highlighting the need for more research to clarify the role of this polymorphism in athletic performance. The absence of a significant relationship between power athlete status and this polymorphism in our study may also be influenced by the characteristics of the studies included in this meta-analysis. Factors such as differences in sports disciplines, gender, and ethnic backgrounds of the participants could have affected the findings. Therefore, it is important to note that these results do not definitively exclude a potential association between this genetic polymorphism and power athlete status. Such factors could influence both the findings of the study and the role of the related polymorphism in athlete status. For instance, in the study by Yvert et al. [62], sprint/power athletes and control groups included participants from different ethnic backgrounds, including a Japanese population. In contrast, Mustafina et al.'s study incorporated Russian and Polish endurance athletes. Additionally, considering that gender may modulate the effects of genetic variants, the absence of a significant difference in women suggests that this factor should be considered in the analysis of genetic polymorphisms and their impact on athletic performance. Considering that gender can modulate the effects of genetic variants, the lack of a significant difference observed among women highlights the potential role of sex-specific biological factors [70]. Sullivan's research emphasizes that the renin-angiotensin system exhibits differential responses to stimulation and inhibition based on gender. This indicates that genetic polymorphisms can affect athletic performance differently in males and females.

When reviewing the literature, it is observed that there are studies revealing the association between the *FTO A/T* (*rs9939609*) polymorphism and power athlete status. In their study, Guilherme et al. [34] found that the frequency of the *A* allele was higher in power athletes, particularly those in the heavyweight category, compared to endurance athletes. In addition, a decrease in the proportion of slow-contracting muscle fibers was observed in power athletes carrying the *A/A* genotype. Initially known as a risk gene for increased body weight, recent research suggests that the *FTO* gene is not only associated with an increase in adipose tissue mass but also with having more lean mass [31, 71]. This, especially the increase in lean body mass, can be considered as an advantage in terms of anaerobic performance. However, when other studies are examined, no significant relationship has been observed between the *FTO A/T* polymorphism and power athlete status [36, 53]. So far, it has been observed that there is only one meta-analysis study determining the relationship between the relevant gene and power athlete status. In the meta-analysis conducted by Weyerstraß et al. [2] similarly, no significant difference was found. In our study, when comparing the genotype and allele distributions between power athletes and the control group, no significant differences were observed in either genotype or allele distribution frequencies. This situation may be attributed to the limited number of studies included, as well as the insufficient understanding of the biological context linking the *FTO A/T* (*rs9939609*) polymorphism to power athlete status. Although another study reported a higher frequency of the minor (*A*) allele in power athletes, associating it with greater total body mass and a lower proportion of slow-twitch muscle fibers [34], a previous study found that the major (*T*) allele was linked to greater lean mass and a leaner body composition [71]. Additionally, while a homogeneous allele (p > 0.05; I^2^ = 0%) distribution was observed in this study, investigating the relationship between this polymorphism and power athlete status requires larger sample sizes for more robust conclusions.

After reviewing the existing literatüre, no meta-analysis study related to the *GALNTL6 C/T* (*rs558129*) polymorphism has been encountered. According to the results of this meta-analysis, the frequency of the *C* allele [OR: −0.30 (−0.48; −0.12), p = 0.001] and the *C/C* genotype [OR: −0.32 (−0.55; −0.09), p = 0.005] was significantly higher in the control group compared to power athletes. In contrast, a higher frequency of the *T/T* genotype [OR: 0.54 (0.11; 0.96), p = 0.014] was observed in power athletes compared to the control group. When looking at the studies, even though we don't fully understand the specific role of *GALNTL6 C/T* (*rs558129*) polymorphism in human biological mechanisms, it has been linked to factors related to adapting to physical activity, gut microbiota, and processes related to energy production, storage, and expenditure by these bacteria [72]. Catalyzed by the *GALNTL6* enzyme, O-glycosylation plays a crucial role in important functions such as the conversion of gut microbiota glycans to short-chain fatty acids (SCFA), immune system regulation, anti-inflammation, and metabolism control. These SCFAs have been associated with increased fitness and overall health in athletes compared to sedentary groups [72, 73] Especially considering that this gene may influence energy metabolism, it can be speculated that it might be associated with anaerobic performance. In a study conducted by Díaz Ramírez et al. [40] it was observed that the distribution of the *T* allele in the *GALNTL6 C/T* polymorphism was higher in power athletes compared to both control and endurance athletes. Moreover, compared to *C/C* genotype carriers, individuals carrying the *T* allele exhibited higher power values in the Wingate Anaerobic Test (WAnT). Our study, in addition, is supported by a study conducted by Zmijewski et al. [37] that yielded similar results. Considering the findings of this study, although minimal heterogeneity was observed in the analysis results (p < 0.05; I^2^ = 0.76%), publication bias was also detected among the studies (p = 0.01). This suggests that while the meta-analysis proposes a potential significant relationship between power athlete status and the *GALNTL6 C/T* (*rs558129*) polymorphism, further research is needed to support these findings and draw more conclusive results.

Another gene that reveals the relationship between genetic polymorphism and athlete status is the *GNB3 C825T* polymorphism. When studies are examined, statistically significant relationships in particular with endurance athlete status have been observed in the *GNB3 C825T* (*rs5443*) polymorphism [45, 46]. In the study by Eynon et al. [46], it was found that the distribution frequencies of the *T/T* genotype were higher in endurance athletes compared to both control and power athletes. In contrast to these studies, there are others that did not find a significant relationship between athlete status and *GNB3 C825T* polymorphism [44, 63, 64]. In another study, although the *T* allele frequency was previously associated with endurance athlete status, Yvert et al. [74] found that the distribution frequency of the *C* allele was higher in endurance athletes compared to the control group. Upon reviewing the literature, in addition to the contradicting nature of these studies, it has been observed that there is only one meta-analysis study examining the relationship between *GNB3 C825T* polymorphism and power athlete status [2]. When examining the findings of the study, no significant relationship was found between *GNB3 C825T* polymorphism and power athlete status. Similarly, upon reviewing the findings of our study, no significant differences were observed in the genotype and allele distribution frequencies of *GNB3 C825T* polymorphism between both control and power athletes. Additionally, the analysis results indicate that there is no evidence of heterogeneity (p > 0.05; I^2^ = 0%), or publication bias (p = 0.210) among the studies. This finding suggests that the studies demonstrate consistent and homogeneous distribution frequencies. However, considering the complex physiology of genetics, it is plausible that both alleles of a single polymorphism may be associated with athlete status, as suggested by previous findings. For instance, in the well-known *ACE I/D* polymorphism, the *I* allele has been linked to endurance athlete status, while the *D* allele has been associated with power athlete status [2, 16]. Moreover, studies often focus on single SNPs when investigating the genetic factors influencing athlete status. While this approach helps understand the role of a specific polymorphism, it may be insufficient to fully explain its relationship with athletic performance. In contrast, SNP-SNP interactions may provide broader biological insights and a more comprehensive understanding of complex traits like athletic performance. For example, a study found that individuals with the *ACTN3 R/R* genotype combined with the *ACE I* allele had a 2.67-fold higher likelihood of being power athletes compared to controls (OR: 2.67; 95% CI: 1.45–4.93) [75]. A recent study investigated the combined effect of 28 polymorphisms potentially associated with power athlete status. In this study, all athletes were classified based on the number of "power" alleles they carried. The results revealed that highly elite power athletes possessed at least 22 "power" alleles, whereas the control group had fewer than 22. Similarly, the proportion of individuals carrying a high number of "power" alleles was significantly higher in highly elite power athletes (84.8%) compared to less successful power athletes (64.9%) [76]. Although the findings of this study provide a general perspective on the potential association between the *GNB3 C825T* polymorphism and power athlete status, it is important to acknowledge the significance of polygenic influences, particularly in sports that require high performance. This complexity may lead to varying results regarding the role of the gene in question.

The increased awareness of the importance of explosive power and anaerobic metabolism in individuals involved in power sports has led to various studies examining the effects of genetic diversity on these physiological characteristics [3, 77]. Another candidate gene thought to be associated with athlete status and genetic polymorphism, which could potentially impact physiological characteristics, is *MCT1 T1470A* (*rs1049434*). In the study conducted by Sawczuk et al. [66] the *MCT1 T1470A* polymorphism's genotypic and allelic distribution frequencies were examined in power athletes and sedentary individuals. According to the study results, it was concluded that power athletes have a higher frequency of the *T* allele and *T/T* genotype compared to the control group. When another study is examined, a high frequency of the *A* allele and *A/A* genotype was found in endurance athletes compared to the control group, while a high frequency of the *T* allele was observed in power athletes [49]. Physiologically, changes in individuals carrying the *T* allele are suggested to increase blood lactate concentration by reducing lactate oxidation, thereby triggering muscle fatigue [49, 74]. When examining the findings of this study, similar results were identified. Although no significant difference was observed in major allele (*T*) and recessive model in *MCT1 T1470A* polymorphism, it was observed that the distribution frequency of the *T/T* genotype [OR: 0.34 (−0.10; 0.59) p = 0.006] was higher in power athletes compared to the control group. In the meta-analysis study conducted by Weyerstraß et al. [2], which addressed the relationship between 9 genetic polymorphisms and power athlete status, no significant relationship was observed between *MCT1 T1470A* polymorphism and power athlete status. It can be suggested that the significant difference found in our study may be due to reaching a larger sample size. Moreover, while a significant difference was observed in the dominant model (*T/T*), the absence of significance in the major allele (*T*) could potentially be attributed to high heterogeneity (p > 0.05; I^2^ = 76.77%). However, an important point that needs to be emphasized is that in the study by Dzitkowska-Zabielska et al. [48] the *MCT1 T1470A* polymorphism was considered the *T* allele as the major allele. While other studies, such as the one by Fedotovskaya et al. [49] have classified the *T* allele of the MCT1 T1470A polymorphism as the minor allele, in our study, the *T* allele was considered the major allele based on population frequency data obtained from the Ensembl database (https://www.ensembl.org/Homo_sapiens/Variation/Population?db=rs1049434;vdb=variation).

This study, lastly, examined the allele and genotype distribution frequencies of the *NOS3 786 T/C* (*rs2070744*) variant, which is considered to play a significant role in genetic polymorphism and power athlete status and is supported by other studies [1, 78]. According to the findings of the study, it was determined that the distribution frequencies of the *T* allele [OR: 0.50 (0.07; 0.93) p = 0.022] and *T/T* genotype [OR: 0.73 (0.12; 1.34) p = 0.018] of the *NOS3 786 T/C* polymorphism are higher in power athletes compared to the control group. No significant difference was found in the comparison of the two groups in the recessive model. Based on these findings, it can be considered that having the *T* allele and *T/T* genotype may be an advantage for power athletes. In addition to what was mentioned above regarding NO, NO, which serves as a vasodilator, plays a crucial role in regulating blood flow to tissues containing actively working muscles, especially [79]. Sessa et al. [67] has reached similar results parallel to the findings of our study. According to their findings, the *NOS3 786 T/C* polymorphism showed a higher distribution of the *T* allele in power athletes. Similar results were observed in the study Drozdovska et al. [68]. When examining the studies, it is demonstrated that moderate and high-intensity exercises increase NO production [80]. Particularly, considering the effects of Nitric Oxide (NO) on vascular tone, it should be noted that it might play a crucial role in transporting more oxygen and nutrients to the muscles, aiding power athletes in enduring longer during workouts. According to the results of this study, the *T* allele frequency of the *786 T/C* polymorphism in the *NOS3* gene responsible for NO production is thought to be positively associated with power athlete status. However, upon reviewing the analysis results, high heterogeneity (p < 0.05; I^2^ = 73.73%) and publication bias (p = 0.001) were observed. It is suggested that the high heterogeneity may stem from factors such as the sample size or population characteristics of the included studies [81]. Specifically, the inclusion of studies with small sample sizes could contribute to a broader prediction interval and result in more variable and random outcomes.

## Conclusıons

In recent times, genetic polymorphism, which has become increasingly important in athlete status, represents various variations among individuals. This study examined certain genetic polymorphisms potentially associated with power athlete status through meta-analysis, comparing control and athlete groups. The findings of the study indicate that among the six genetic polymorphisms (SNPs) examined, *GALNTL6* (*rs558129*), *MCT1* (*rs1049434*), and *NOS3* (*rs2070744*) polymorphisms may be associated with power athlete status. In the genotype and allele distribution results, it was observed that the *GALNTL6* T/T genotype, *MCT1* T/T genotype, and *NOS3* T allele and T/T genotype frequencies were higher among power athletes. Based on these meta-analysis results, it is suggested that these polymorphisms may play a significant role, particularly in power athlete status, and further research support may be needed to explore their significance.

In summary**,** the genetic polymorphism, identified as a significant factor influencing athlete status, contributes significantly to the literature through these findings. Especially when considering the genetic predisposition underlying physiological mechanisms and playing a role in different athlete statuses, differences in performance should be taken into account. Determining the distribution of genetic polymorphisms that could contribute to athlete status can help athletes optimize their training and performance strategies more effectively. However, this can only be supported by existing and future research. Based on these findings, collecting data and conducting robust studies such as systematic reviews and meta-analyses can provide readers with a general perspective, potentially pioneering future applications. While the studies included in this meta-analysis may offer valuable insights, further qualitative and quantitative research is needed to assess the relationship between power athlete status and genetic polymorphism comprehensively.

## Limitations of the study

Considering the limitations of this study, a limited number of studies were included in the meta-analysis to assess the role of genetic polymorphisms in power athlete status. Each polymorphism also had a limited number of studies, and subcategories such as gender, sport discipline, and ethnicity were not evaluated separately, which represents one of the limitations of the study. The inability to perform these evaluations was due to the insufficient data available for calculating Odds Ratios (ORs). Another limitation of this study was the inclusion of only English-language articles and original research papers. Regarding the strengths of the study, PubMed and Web of Science databases were used with the aim of enhancing the reliability and quality of the research. Furthermore, the quality of the included studies was assessed using the Newcastle–Ottawa Scale (NOS)

## Data Availability

The data supporting the findings of this study are accessible upon reasonable request from the corresponding author.
